# Multicellularity in animals: The potential for within-organism conflict

**DOI:** 10.1073/pnas.2120457119

**Published:** 2022-07-21

**Authors:** Jack Howe, Jochen C. Rink, Bo Wang, Ashleigh S. Griffin

**Affiliations:** ^a^Department of Zoology, University of Oxford, Oxford OX1 3SZ, United Kingdom;; ^b^Center for Evolutionary Hologenomics, Globe Institute, University of Copenhagen, Copenhagen 1353, Denmark;; ^c^Max Planck Institute for Multidisciplinary Sciences, 37077 Göttingen, Germany;; ^d^Department of Bioengineering, Stanford University, Stanford, CA 94305;; ^e^Department of Developmental Biology, Stanford University School of Medicine, Stanford, CA 94305

**Keywords:** evolution, multicellularity, development

## Abstract

Metazoans function as individual organisms but also as “colonies” of cells whose single-celled ancestors lived and reproduced independently. Insights from evolutionary biology about multicellular group formation help us understand the behavior of cells: why they cooperate, and why cooperation sometimes breaks down. Current explanations for multicellularity focus on two aspects of development which promote cooperation and limit conflict among cells: a single-cell bottleneck, which creates organisms composed of clones, and a separation of somatic and germ cell lineages, which reduces the selective advantage of cheating. However, many obligately multicellular organisms thrive with neither, creating the potential for within-organism conflict. Here, we argue that the prevalence of such organisms throughout the Metazoa requires us to refine our preconceptions of conflict-free multicellularity. Evolutionary theory must incorporate developmental mechanisms across a broad range of organisms—such as unusual reproductive strategies, totipotency, and cell competition—while developmental biology must incorporate evolutionary principles. To facilitate this cross-disciplinary approach, we provide a conceptual overview from evolutionary biology for developmental biologists, using analogous examples in the well-studied social insects.

Life is hierarchically organized (1): Genes cooperate to form genomes, discrete genomes to produce eukaryotic cells, and millions of cells to produce a human or an oak tree. In each case, lower units formed a cooperative group and lost their ability to reproduce independently—each underwent a major evolutionary transition (1, 2). This raises a new question: Cooperative groups are vulnerable to the “selfish” interests of the individuals that comprise them, so how is cooperation maintained? (3) In a population of cells comprising a multicellular organism, selection might favor cells that neglect producing soma to increase investment in reproductive germline (4–6) or that selfishly proliferate as cancers (see Box 1). While the potential consequences of within-organism selection have been discussed widely (e.g., refs. 7–12), the technologies required to test these ideas have only recently become available and developmental biology has therefore recently begun to incorporate them. Testing these ideas, however, requires a cross-disciplinary approach built on a common foundation.

Box 1. **Are Cancers Cheats?**

Cancers represent the breakdown of cooperation in multicellular organisms: Mutations allow cells to escape the brakes on their division, to overproliferate, and to disrupt organismal function with often fatal consequences for the organism. Cancerous cells, therefore, are often considered cheats (71, 73).

Cancerous cells differ fundamentally, however, from cheats observed in cooperative groups more generally. Unlike workers that lay eggs in social insect colonies (98), bacterial cells that fail to contribute to public goods (106), or bees that steal nectar without pollinating (107), cancer cells have no reproductive future. While we expect natural selection to operate within organisms to some degree, and that this might lead to selection to overproliferate, evolution within cancers is restricted because:

1) it is a brief bout of natural selection, which cannot accumulate over evolutionary time. While cancer cell lineages may show simple adaptations—such as adapting to different environments within a tumor or increased metastasis—this process is short-lived and prevents selection from producing complex adaptations like eyes (29, 108). Cancer cells could be described as “short-sighted” cheats that are favored within a group but cannot transmit to future generations—like mutant viruses which flourish within-host but cannot transmit between hosts (109).
2) tumors vary greatly through time and space, so selection is aiming for a target that shifts too rapidly for generations of cells to adapt to, rather than in a consistent direction.
3) cancer cells are (generally) restricted to a single host, preventing mutants from sweeping through the population.

The fragmented and inconsistent selection among cells within an organism’s lifespan is in contrast to the selection acting on genes inherited through the germline for many generations (18). Longer-term selection allows for complex adaptations, like defenses against cancers, and means that tumors are a mirror image of those defenses (95). The body will have adapted to the probabilities of different tumors arising, their costs, and the cost of the defenses against other uses of energy such as growth, reproduction, or defense against infectious disease. Organisms will have cellular machinery mutating and generating cancer where those defenses are weakest, but there is no cleverness in the way cancers arise and evolve. For this reason, cancerous cells differ from “cheats” in multicellular systems we discuss in this review. A cell that escapes the fate of the organism in which it originates, by transmitting to new generations or organisms, can be honed by natural selection. Interestingly, some cancers do just that: Transmissible cancers are found in dogs (110), Tasmanian devils (111), and clams (112) and show adaptations to avoid host defenses (110). Evolutionary theory provides us with the tools to understand and predict the prevalence and nature of cheating in different scenarios (3, 71).

Here, we provide this common foundation: an introduction to current thinking in evolutionary biology on multicellularity for developmental biologists and a guide to relevant aspects of developmental biology for evolutionary biologists. We assess whether our understanding of cellular, developmental, and reproductive biology across the Metazoa confirms or challenges expectations from evolutionary biology and highlight avenues of future research. While we concentrate on animals, this discussion applies to all multicellular groups (see refs. 12 and 13 for previous treatments of intraorganismal conflict). These discussions apply most strongly to “unitary” organisms rather than “modular” organisms built from physically connected units that are at least partially self-sustaining and able to reproduce, as in tree branches or coral polyps. The complete interdependence of parts in a unitary organism means they survive and reproduce as a whole (9) (for discussions of modular organisms see refs. 12 and 13). We provide a background of the relevant theory, which generates clear expectations of the conditions required for multicellularity to evolve and persist. We then explore the biology of some of the many animals that defy these expectations. Here, we apply a major evolutionary transitions framework to understand the evolution of multicellularity (see refs. 14–17 for reviews with a more mechanistic perspective). We use the analogous transition to sociality in insects, arguably the best-studied major transition, to highlight where our understanding of multicellular evolution is lacking. As is common in evolutionary literature, we use “intentional” language like “selfish” (*SI Appendix*, *Glossary*).

## The Evolution of Multicellularity

Multicellularity has arisen independently at least 25 times (18). The majority of multicellular groups, however, are simple facultative aggregations: Each cell can survive and reproduce independently and can establish another multicellular group (18). Obligate multicellularity is rarer, seen in animals, plants, fungi, red algae, brown algae, and potentially some ciliates and cyanobacteria (19, 20). Under obligate multicellularity, cells cannot survive or reproduce independently of the multicellular group, and groups contain multiple sterile cell types (19). While cells in obligately multicellular organisms still divide, only a fraction can establish a new group, so this within-organism division is not considered reproduction.

Two conditions are often described as necessary for the evolution of obligate multicellularity: development from a single cell (1, 19, 21) and an early and strict separation of sterile somatic cells from reproductive germ cell lineages (7, 22). These mechanisms align all cells’ fitness interests and are predicted to minimize conflict within multicellular groups, by creating a clonal group with limited access to future generations.

### Single-Cell Bottlenecks and Clonality.

Multicellularity is cooperative: Cells sacrifice reproducing independently to reproduce as a group. Genes encoding altruistic traits that reduce reproduction, like sterility, can only be favored if fitness costs are outweighed by fitness benefits to relatives that carry that same gene (23, 24). Relatives carry the gene with a particular probability (i.e., relatedness; Box 2), so the fitness benefits, weighted by this probability, must exceed the cost to the altruist. The lower the relatedness, the greater the benefit must be for a given cost for a gene to spread. In clonal groups, all members carry identical genes, so benefits to relatives must simply exceed the costs. The fitness interests of group members are therefore perfectly coincident: So long as a trait increases group reproduction, it is favored. This theory then predicts that cells will sacrifice their own reproduction to increase group reproduction (24).

Box 2. **Relatedness**

Relatedness is vital for our understanding of multicellularity: Without positive relatedness between individuals, altruistic traits cannot evolve. Relatedness describes genetic similarity. It expresses the probability that individuals share a gene identical by descent (23, 24): Intuitively, there is a 50% chance that two full siblings received the same gene copy from their parents in a large, randomly mating population. Relatedness (*r*) is vital for understanding the evolution of social behavior; it tells us when to expect altruism and self-restraint (high *r*), selfish exploitation and conflict (low *r*), or spite (negative *r*)—depending on a trait’s costs and benefits (113). The importance of relatedness has repeatedly been vindicated: when social insect workers should lay eggs and when other workers should remove them (98), the sex ratio of reproductives produced by insect colonies (114), the production of public goods in bacteria (106), and helping behavior in birds (115), among others. Where cells are clonally related, as in multicellular organisms starting from a single cell, any gene in one cell is certain to be in any other (excepting rare mutations) and highly altruistic traits like sterility can evolve (1, 19).

The relevant relatedness coefficient, however, is not across the whole genome but only at the locus that controls the trait under consideration (23). Usually, we do not know which loci encode interesting traits, so estimates of relatedness have often considered average, whole-genome relatedness values, e.g., in groups of *Dictyostelium* amoebae (33) or *Polistes* wasps (93).

Typically, genome-wide estimates suffice, as relatedness is highly correlated across the genome (116) and the fitness interests of most genes are aligned for many traits (but see ref. 88). Mutations, however, highlight the importance of locus-specific relatedness: A mutation creates a gene not found in other group members (i.e., *r* = 0), and selection will act accordingly (24). The spread of cheating lineages in *Pseudomonas* populations in chronic lung infections demonstrate this (117): Mutations that disrupt the secretion of iron-scavenging public goods rapidly spread since mutants benefit from the cooperative secretions of others without paying a cost themselves (106). Two cells can be almost genetically identical, but the few differences caused by mutation can cause significant conflict.

**Fig. 1.**
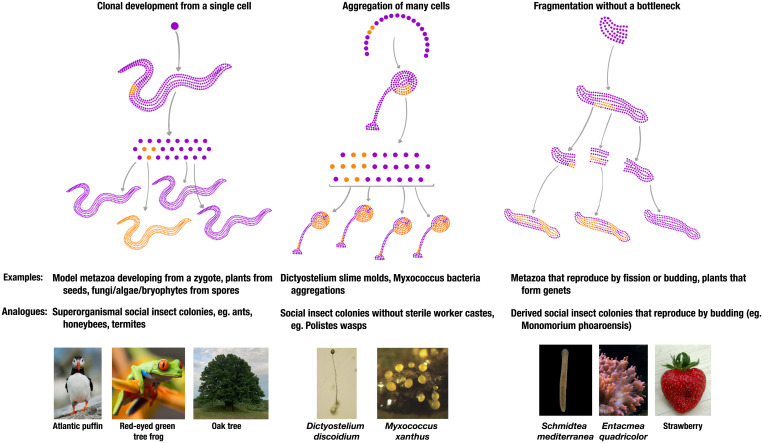
Clonal development and single-cell bottlenecks. Clonal development from a single cell ensures all cells within a multicellular organism are clonally related, thereby facilitating cooperation. If mutations (orange) arise they are segregated in the next generation and offspring remain clonal. In aggregative groups, cells may not be clonally related, and neither are any subsequent groups produced—selfish mutants (orange) that preferentially contribute to reproduction can spread within the population under the right conditions. When groups develop clonally from large propagules (e.g., fission), mutations may not be segregated among offspring but may persist through generations. This could select for selfish cell lineages that could not otherwise spread. See ref. 118 for a similar figure discussing bacterial colonies. Image credit: Unsplash: Ray Henessy, Stephanie LeBlanc, Ivan Matveev; Wikimedia Commons: Michael Vos, Bruno in Columbus, Vassil, Paolo Neo; Uri Weill, Rink laboratory, Max Planck Institute.

A single-cell bottleneck is perhaps the simplest way to establish a clonal group: One cell dividing clonally produces an organism comprising genetically identical cells with perfectly aligned interests (Fig. 1) (11, 25). Multicellular organisms will not remain perfect clones, however, as mutations inevitably arise during development. Mutation creates variation that selection can act upon, causing a divergence of fitness interests within the group. Most mutations are likely deleterious for cell function so will be removed by within-organism selection among cells (9, 10, 26). Occasionally, however, mutations may be beneficial at the cell level but harmful to the multicellular organism. Here, selection will act in opposing directions among cells and among organisms. Cancers provide a dramatic demonstration: Mutations cause replicative advantages that are favored in selection among cells but are often fatal to the organism (7, 27).

Regular single-cell bottlenecks also reset within-group clonality and realign group-member fitness interests. They segregate genetic variance among offspring and shift selection from among cells to among clonal multicellular groups (21, 25, 28, 29): A group started from a mutant uncooperative cell will comprise only uncooperative cells, so will be selected against compared to groups of cooperators (18, 21, 29, 30). Frequent single-cell bottlenecks therefore limit the potential for selfish cell lineages to persist beyond an organism’s lifespan. A single-cell bottleneck may bring additional benefits by increasing developmental flexibility: Development starts afresh each generation rather than building on a complicated body plan, and all cells inherit any evolutionary innovations (25, 31).

Many multicellular organisms, however, do not develop clonally from a single cell. Some groups form by aggregation. Compared to groups formed by clonal expansion, aggregative groups are likely more genetically variable and therefore have greater potential for internal selection and conflict (3, 21). *Dictyostelium* cellular slime molds demonstrate the consequences of within-group variation and selection: their facultative multicellular groups form through aggregation of amoeboid cells to build a fruiting body to aid dispersal (32). In *Dictyostelium discoideum*, aggregating cells have an average relatedness of 0.98 in natural populations (33). Positive relatedness means that altruistic traits—like the sterile stalk in fruiting bodies—are favored. However, relatedness is not clonal (*r <* 1), cells’ interests are not perfectly aligned, and selfish traits can spread, like preferential development into reproductive spores (33). Selfishness leads, in turn, to selection for suppression of competition, such as genetic sorting mechanisms (34). However, such mechanisms are unlikely to suppress conflict so completely as to enable an evolutionary transition to obligate multicellularity (2, 35), and *Dictyostelium* remains facultatively multicellular (32). Complex, obligate multicellularity with multiple sterile cell types only evolved in groups formed clonally through cell division (19).

Further, some obligately multicellular organisms that do form through clonal cell division do not always start from a single cell. Instead, they often reproduce by budding or fission, with propagules containing potentially thousands of cells. In the presence of mutation, propagules must be small to maintain clonality (36) and the large propagules observed in many animals could allow genetically heterogeneous groups to arise and persist (Fig. 1) (12). Many Metazoans can fission or bud, although most phyla employ both sexual and fissiparous reproduction strategies, often within the same species (see Fig. 3) (14). In the Cnidaria, *Hydra* typically reproduce by budding off new individuals (37), while many anenomes can split and regenerate missing tissues (38). The same applies in many bilateria: Acoelomorpha worms can fission, as can many sea stars and sea cucumbers (39), and annelids (40) and Platyhelminthes (41) can both bud and fission. While each phylum contains species with regular strict, single-cell bottlenecks [e.g., leeches in the Annelida (40)], fission or budding is observed in all phyla, suggesting it may be ancestral to Metazoa (14). Wherever organisms utilize fission or budding, many asexual generations without single-cell bottlenecks may separate rarer sexual generations, enabling long bouts of within-organism selection (27).

Metazoan reproductive strategies span a continuum from reproduction with a single-cell bottleneck every generation to exclusively fissiparous reproduction without bottlenecks. Their position on this continuum and the number of cell divisions between bottlenecks determines the potential for mutations, and therefore selection and conflict (5, 27)—even rare bottlenecks would reset group clonality and may prevent conflict (5). At one extreme, organisms like humans or *Caenorhabditis elegans* pass through a bottleneck every generation. Some species alternate between sexual reproduction and fission: Parasitic schistosomes proliferate asexually in their molluscan intermediate hosts but produce eggs in mammalian hosts (42). Others have prolonged periods of asexual propagation, with sexual reproduction triggered by intrinsic or extrinsic factors: *Hydra* buds off asexual individuals every few days, with occasional sexual reproduction in response to environmental conditions or population density (43).

At the fissiparous extreme, some organisms apparently never pass through a single-cell bottleneck. Exclusively fissiparous species appear rare, perhaps because we have simply not observed them and there are more than currently known, but likely because the short-term benefits of asexual reproduction through fission are negated over longer timescales. Asexual organisms can rapidly propagate but may be less able to adapt to changeable conditions or diseases, or may be susceptible to internal selection among selfish cells. Asexual strains of the planarian flatworm *Schmidtea mediterannea* have been maintained in laboratories for decades and reproduce exclusively by fission, with genetic rearrangements that appear to prevent meiosis (44). These asexual worms seem to function as well as sexual strains, despite increased potential for selection and conflict among cells. Unfortunately, we know little of the relative rates of sexual and fissiparous reproduction in nature—even apparently obligate asexuals may have rare, unobserved sex. Many cell divisions between bottlenecks may be required for selfish cell lineages to persist (5), yet comparing mutational load between obligately sexual and fissiparous planarian populations suggests that fissiparous organisms have increased potential for within-organism selection: Individuals from asexual populations carried more mitochondrial diversity than those from sexual populations (45). Clonality through a single-celled bottleneck is far from universal across animals.

### Germline Segregation.

The specialization of cells into reproductive germline versus sterile soma represents the most fundamental division of labor in obligately multicellular organisms. Differentiating into a somatic cell is altruistic: A somatic cell sacrifices its immortality, relying on germ cells to transmit their genes to future generations. Sterility can only evolve if there is a net gain in gene copies produced, so somatic and germline cells must be closely related (Box 2) (23).

Benefits from the separation of reproductive and somatic roles are illustrated by the obligately multicellular volvocine algae (46). In the volvocine algae, cells cannot reproduce and remain motile simultaneously for long (47). Therefore, in volvocine species without differentiated cells, like *Gonium*, all cells perform all tasks and must switch between them, limiting group size and complexity (46, 48). *Volvox,* however, avoids this by specializing some cells in motility and others in reproduction, enabling both to occur simultaneously, and facilitating larger groups with more cell types (47, 49).

Once a reproductive division of labor has evolved, a strict and early separation of somatic and reproductive functions may enable further complexity (22, 50). Early germline sequestration may increase developmental flexibility as cells need not maintain a prolonged pluripotent state (51). Alternatively, but not mutually exclusively, sequestration may reduce the potential for reproductive conflict (7, 22). If somatic differentiation is irreversible, somatic cells can only gain fitness through helping related germ cells (22, 50). Even if some somatic cells still divide (52), somatic mutations are still lost upon organismal death as they cannot enter the germline.

**Fig. 2.**
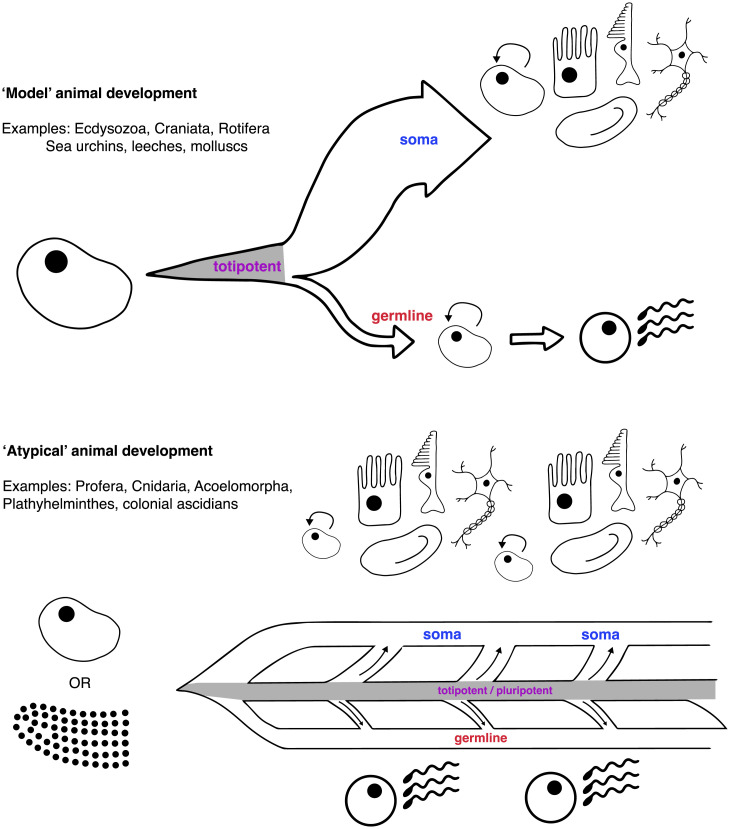
Germ–soma segregation. The relationships between the cells during multicellular development. Under strict germ–soma separation, the germline and soma are separated early on, cells maintain totipotency (gray) for relatively few cell divisions, and no pluripotent cells are present in the adult. This is predicted to prevent selfish mutants arising and disrupting cooperation, but many animal groups appear to have no separation. In these animals, a subset of cells contributes to germline and soma even as adults (gray). Totipotency must be maintained, potentially creating conflict.

The timing of mutations in development is crucial: The earlier the germ and soma separate, the smaller the window for selfish mutants to arise within the germline (Fig. 2) (22). Buss suggested that early germline segregation is a direct manifestation of conflict among cells (7, 8): If a mutation that caused earlier germline differentiation were to arise before germline segregation, offspring would comprise “early-germline” cells, provided there are no deleterious pleiotropic effects of the mutation and that early germline segregation itself is not too deleterious. The first metazoans, however, likely contained few cells, so the chance of mutations arising before reproduction was small and a strict germline may not have been required (53). Thus, while a reproductive division of labor likely arose because of productivity benefits, selection for reduced conflict might have shifted germline segregation earlier (54), potentially enabling the increased complexity observed in many animal lineages (7, 55).

Many model species segregate their germline early in development (14–16). In *C. elegans*, the two primordial germ cells are demarcated after four cell divisions, marked by maternal factors asymmetrically deposited in the oocyte (56). In sea urchins, germline cells are demarcated between the third and fourth cell division, when asymmetrical cell divisions create two small micromeres that will form the germline (57). In vertebrates, germline specification occurs shortly before gastrulation in mice (∼6.5 d postfertilization) (58) and several weeks postfertilization in humans (59). While late relative to *C. elegans,* this is early relative to reproductive maturity, when these cells are needed. Compelling demonstrations of strict germline segregation are provided by the germline’s inability to be replaced if experimentally ablated: When the germline progenitors in a 16-cell sea urchin, or an eight-cell *C. elegans* embryo are removed, development proceeds normally, but the gonads formed lack gametes (56, 60). Likewise, after some ambiguity (e.g., ref. 61), experiments have confirmed that the germline cannot be replaced in mice (62), while human females are born with their lifetime complement of oocytes (63).

**Fig. 3.**
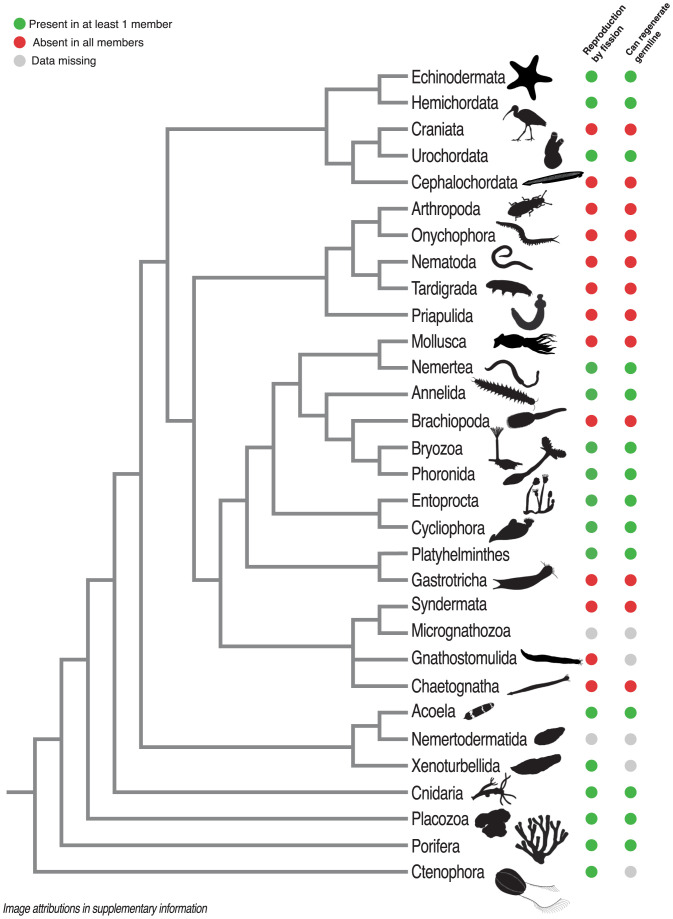
Phylogenetic distribution of fissiparous reproduction and germline regeneration. A phylogeny of the metazoan phyla with the presence or absence of traits predicted to generate conflict within an organism: the ability to reproduce asexually through propagules containing many cells (i.e., fission or budding), and the ability to regenerate the germline if it is physically or chemically removed as an indicator of pluripotent cells. Green circles indicate the presence of a trait in any member of a phylum, red indicates where no members possess a trait, and gray indicates a lack of data. Data predominantly from refs. 14, 15, 17, 69, and 119. Image credit: Phloypic: Michelle Site, Mali'o Kodis; Wikimedia Commons: Snoteleks; Flickr: Derek Keats.

Many animals, however, do not segregate a germline (Fig. 3) (14, 15, 17). In the nonbilaterian animal lineages, pluripotent cells that contribute to both germline and soma throughout life are common (64): Sponges have archeocytes (65), and *Hydra* have “interstitial cells” (37). Pluripotent cells also occur in many bilaterial lineages: the neoblasts of the aceolomorpha and the planarian flatworms (Platyhelminthes) (66, 67), or the blood-borne stem cells of colonial ascidians (Chordata) (4, 68). Some animals can regenerate gonads and gametes when removed, implying the presence of pluripotent cells (69); in the Annelida, a worm produced by asexual fission that lacks gonads can still develop the germline (70), and some starfish (Echinodermata) can regrow gonads (although sea urchins apparently cannot) (60). Only few groups have a strict, early segregation (Mollusca, Ecdysozoa, Chaetognatha, Rotifera, Micrognathozoa, and Craniata, although this may change with more data), implying that the ancestral Metazoan did not (14, 15, 17). Broadly throughout the Metazoa, therefore, the window for mutations to arise and enter the germline never closes (15), and the potential for reproductive conflict before germline segregation may be greater than often assumed.

## Cell-Level Selection within Organisms

Based on the evolutionary theory above, a multicellular organism is expected to start from a single cell, which divides to produce clonally related daughter cells. Early in development, a subset of cells are demarcated as the future germline, while the remaining cells differentiate into a diversity of sterile somatic cells—perhaps still capable of division, but restricted to produce only somatic cells. Upon reproductive maturity, single cells derived from the germline progenitors disperse to start the next generation. In short, they resemble us, or many of our model organisms: *C. elegans*, *Drosophila melanogaster*, *Danio rerio*, *Xenopus laevis*, or *Mus musculus.* Yet, as highlighted, many organisms defy these expectations to varying degrees. The potential for within-organism selection, and therefore conflict, is the norm, not the exception.

Conflict within organisms may manifest in several ways. Typically, cells cooperate by sacrificing their own reproduction or survival for the good of the organism: They apoptose or cooperatively develop down the evolutionary “cul-de-sacs’ of somatic lineages over “immortality” through the germline. Cells with selfish mutations, however, might parasitize these cooperative behaviors by overproliferating as cancers (Box 1) or remaining pluripotent (3, 71, 72). Yet, despite the possibility for within-organism selection, overt conflict among cells within multicellular organisms appears rare. What is the evidence that organisms with the potential for cellular conflict suffer the predicted consequences?

## Evidence for within-Organism Selection in the Metazoa

Since Buss argued for the role of selection among cells in development in the 1980s (7, 8), his arguments have largely been replaced by a major transitions’ perspective that focuses on single-cell bottlenecks eliminating conflict among cells lineages (1). Decades of data have been collected since Buss’ work and in many cases within-organism genetic diversity and selection are observed—at least in the short term. How does this fit with a conflict-free view of individuality in the metazoans, and how do those dynamics differ in organisms without bottlenecks?

Cancers are obvious manifestations of within-organism selection and are observed across multicellularity (Box 1) (73). Cancerous cells proliferate with often fatal consequences for the larger organism—highlighting the short-sighted, disruptive nature of within-group selection (3). The consequences of within-organism selection, however, are not always so clear and there are few examples of noncancerous cheating by cells in complex obligately multicellular organisms. Such cheating has predominantly been observed in organisms that fuse, such as ascidians (68), fungi (6), or red algae (74). Fusion causes a drop in relatedness across multiple genes, so cells may have misaligned interests, which facilitates the evolution of “parasitic” lineages that stop contributing to soma when fused with other lineages (75). In the only animal example—the colonial ascidian *Botryllus*—some strains stop contributing to the soma after fusion and are only present in the bloodborne stem cells and the gonads, parasitizing the cooperative soma of other strains (76).

Normal developmental often involves selection among competing cells (77, 78)—termed cell competition (79)—but this is not conflict. Murine germline progenitors, for example, embark on lengthy migrations within the developing embryo (80), undergo multiple rounds of proliferation, fragmentation, aggregation, and selection (81, 82), and contribute nonrandomly to offspring (77, 83). Likewise, cell competition contributes to normal development *Drosophila* (84), where “higher-quality” cells divide and replace “loser” cells (85). In these processes, cells are in competition, but not in conflict: All competitors “agree” the “best” cells should win, even if they themselves lose. Clonally related cells will sacrifice themselves, through processes like apoptosis, to increase group reproduction.

Noncancerous conflict has also been observed in humans. A number of human disorders termed “paternal age effect” disorders increase in likelihood with paternal age faster than the germline mutation rate alone (86). They arise because of the regular division of male germline stem cells: Mutations that increase proliferation are favored and come to dominate, and so are overrepresented in offspring. Unfortunately, these same mutations also cause developmental disorders in offspring: Responses to growth signals that were adaptive in the germline stem cell environment are inappropriate in a developing fetus (86, 87). These disorders also highlight the role of single-cell bottlenecks in separating “selfish” cheats from cooperators, as all cells in the offspring will carry these mutations (29, 30). It remains possible, but unexplored, that there are many such mutations favored during gametogenesis, but with less obvious effects on offspring.

While the data for germline selection are sparse, they are even rarer in organisms where we might expect longer bouts of selection. In the above examples, bottlenecks in each generation expose cheats to selection, but in organisms like *Hydra* or asexual planarians with extended periods between bottlenecks, mutation and selection within organism has longer to occur. Here, we require data to establish whether genetic diversity is present and to follow the selective dynamics—within and among organisms.

## Lessons from Other Major Evolutionary Transitions

While conflict within organisms is little-studied in animals, the power of the major evolutionary transitions framework lies in its ability to unite our understanding across the biological hierarchy (1, 21). This view highlights how all levels of the biological hierarchy arose from the same evolutionary processes—that complex group-level adaptations evolve when group-members’ interests coincide, but conflicts arise when they do not. It explains why genes often cooperate to build remarkable adaptations, but also why they are in conflict where they have different inheritance patterns (e.g., nuclear vs. cytoplasmic genes) (88). Similarly, the evolution of multicellularity is driven by the same processes as the evolution of eusocial insect colonies (89). We can therefore look there for analogies (21, 53, 89, 90).

Obligate eusocial insects with sterile workers—i.e., “superorganismal” colonies (91)—evolved only under strict monogamy where single females found colonies after mating with a single male and never remate (e.g., ants, corbiculate bees, vespine wasps, and termites) (Table 1) (89, 92). Strict monogamy creates a relatedness equivalence between offspring and siblings in the same way as a single-cell bottleneck—albeit individuals in social insect colonies are usually not clonally related, and relatedness to siblings and offspring is 0.5. In contrast, social insect groups formed by aggregation, as in *Polistes* wasp colonies (93), face the same problem as *Dictyostelium* aggregations. Cooperation and helping is still favored but is often enforced—as in *Polistes*’ strict reproductive hierarchies (94). Here, reproductive conflict has prevented the transition to “superorganismality” and *Polistes* individuals remain facultative helpers (91).

**Table 1.** The colony as an organism

	Examples	Conditions required	Ratio of relatedness to offspring and relatedness to siblings	Presence of sterile individuals	No. of sterile types	Bottleneck as ancestral?	Secondary reductions in relatedness
Obligate multicellularity	Animals, plants, fungi, red algae, brown algae, ciliates, cyanobacteria	Clonal relatedness of cells due to clonal development	1/1 = 1	Yes	1–200+	Unclear: budding and fission widespread	Fusion (e.g., *Botryllus,* various fungi, some red algae).
Superorganismality	Ants, corbiculate bees, vespine wasps, termites	Relatedness equivalence between offspring and siblings due to strict lifetime monogamy	0.5/0.5 = 1	Yes	1–5+	Yes: strict lifetime monogamy in all cases	Polyandry and polygyny widespread

Multicellular organisms and social insect colonies evolved by analogous processes, so share many traits, such as a sterile “soma.” Both evolved where individuals were equally related to siblings and offspring, yet while insect colonies have subsequently evolved low-relatedness colony structures, multicellular organisms have generally not—with only a few cases of organisms fusing.

For the transition to multicellularity to occur, internal conflict must have been suppressed, but we do not yet fully understand the processes involved. What can we learn from other cooperative groups where conflict resolution has been more thoroughly studied?

### Intergroup Competition.

Selection among organisms offers a solution to within-organism selection: Organisms containing selfish cells are eliminated in competition with organisms composed of more cooperative cells, if selection is strong enough (8, 27, 29). Evolution experiments in facultatively multicellular organisms like *Dictyostelium* (32) demonstrate the importance of the relative strength of within- and between-organism selection: Forcing selection to act within multicellular groups allows cheater cell lineages that contribute less to altruistic stalk formation to spread, when they are otherwise selected against under natural conditions because of among-organism selection (5).

Conflict suppressing mechanisms like single-cell bottlenecks and germline segregation shift selection from within to among groups, exposing cheats to selection. Longer-term selection among organisms can select for suppression of conflict between cell lineages within them, such as tumor-suppressor genes that protect us from the consequences of cell-level selection (95).

### Conflict Is Derived.

Superorganismal insect colonies with high potential conflict—colonies with multiple queens, or multiply-mated queens—are derived, arising only after the major transition to “superorganismality” occurred under strict lifetime monogamy (89, 91, 92). Although individuals in a colony are not clonally related and conflict therefore still arises (e.g., over sex ratios and worker reproduction), lifetime monogamy creates similar conditions to clonality: Individuals are equally related to their offspring and to their siblings, and altruistic sterile workers can evolve (19, 89). Sterility then reduces the potential benefits of not cooperating: A sterile worker ant cannot leave and found a functional colony, and so lower relatedness—through multiply-mated queens or multiple queens—can be tolerated (89, 96). A similar pattern may have occurred in the evolution of multicellular groups, where minimal conflict enabled the evolution of obligate multicellularity, which can then tolerate higher potential conflict. However, reproductive fission and pluripotency are observed throughout the Metazoa, suggesting they may be ancestral (Fig. 3) (14, 29) and that cell-level selection and conflict may have been present in the early Metazoa. Likewise, the first Metazoan likely had at least semiregular single-celled bottlenecks and likely contained many fewer cells than contemporary Metazoans, as suggested by Queller (21) and supported by the Choanoflagellates, the sister group to the Metazoa, containing species with small multicellular stages developing clonally from single cells (97). These are predicted to have limited the potential for mutation, selection and therefore conflict in the first Metazoa.

### Selfishness Is under Group Control.

Even in groups with high potential internal conflict, conflict may rarely be realized. Honey bee colonies have relatively low within-group relatedness, as the queen mates with many males: Selfishly reproductive workers might be expected to be common, but there are numerous mechanisms in honey bees that prevent cheating (98). A strict separation between reproductive queens and sterile workers is enforced by rearing larvae as queens in ‘royal’ cells with a diet of royal jelly or as workers in worker cells (99), while any worker-laid eggs are rapidly removed by other workers (100).

Since the major transitions framework assumes that obligate multicellular organisms are largely conflict-free (1–3), we have perhaps neglected a role for similar enforcement during multicellular development. In this view, the restriction of stem cells to defined niches and cancer suppression by the immune system are similar to the enforcement mechanisms in honey bee colonies (52): limiting reproduction, and actively monitoring for “cheating” cell phenotypes like hyperproliferation or inappropriate responses to signals (73). However, while enforcement can reduce conflict, it is unlikely to completely suppress it, as it is necessary for a major evolutionary transition (1, 2, 21, 35, 89).

### Heterogeneity Brings Benefits.

Genetic diversity provides the raw material for selection, increasing evolvability, but heterogeneity may also provide additional benefits. In social insect colonies, increased genetic diversity can increase colony productivity through a more efficient division of labor, or greater resistance against parasites (96, 101–103). Group fusion can provide immediate benefits and is observed in many facultative and obligate multicellular organisms: *Dictyostelium* “slugs” formed from multiple clones are larger and can migrate further than when clones are partitioned among separate slugs (104), potentially unrelated Rhodophyte algae spores can fuse and form larger holdfasts in turbulent waters (74), and increased genetic diversity from the fusion of mycorrhizal fungi may confer individual level benefits (105). Allorecognition mechanisms can increase within-group relatedness by ensuring only related individuals fuse (75), but even with such mechanisms lineages that favor reproducing over contributing to soma occur in ascidians (4) and rapidly evolve in evolution experiments in fungi (6) and *Dictyostelium* (5). Whether within-organism diversity could lead to individual benefits in the Metazoa, or whether the increased conflict is detrimental, requires experimental testing.

## Conclusion

Research into the evolution of multicellularity has focused on facultatively multicellular organisms, such as *Dictyostelium* slime molds, as they enable manipulation of the relative costs and benefits to cells of independent or group living. Yet, those same characteristics that make them good experimental systems may prevent them from evolving obligate multicellularity. Obligately multicellular organisms, like us, can be considered boringly predictable. However, we highlight that our conflict-free expectations are colored by our narrow set of model organisms, that within-organism selection among cell lineages may be more prevalent than often assumed and that, by exploring more broadly across the Metazoa, a broader understanding of multicellularity could emerge.

## Supplementary Material

Supplementary File

## Data Availability

There are no data underlying this work.
