# Supplementary material for: Multicellularity in animals: The potential for within-organism conflict
Source: Proc Natl Acad Sci U S A. 2022 Jul 21;119(32):e2120457119. doi: 10.1073/pnas.2120457119 (PMC9371690; doi:10.1073/pnas.2120457119)
Supplement: Supplementary File [file pnas.2120457119.sapp.pdf]

Supplementary Information for
Multicellularity in animals: the potential for within-organism conflict

Authors:
Jack Howe[1,2], Jochen C. Rink[3], Bo Wang[4,5], & Ashleigh S. Griffin[1]

Corresponding author: Jack Howe
Email: jack.howe@sund.ku.dk

This PDF file includes:

Supplementary text
Attributions for images in text

Supplementary Information Text
Supplementary materials

Glossary

Consistent with the evolutionary literature, we use 'intentional' language throughout this manuscript to describe how cells, individuals, and even genes behave. While terms like as 'selfish', 'cheat', 'altruism' or 'spite' may seem anthropomorphic and imply intention, they are formally justified on the basis that natural selection causes individuals to act with agency: they are selected to act "as if" they were maximising their fitness, and will therefore act as if they were selfish, cheating, altruistic or spiteful (1). They represent a shorthand for well-defined categories of behaviours, so it is important that they are consistently defined (2).

Cheat / Cheating: a trait that brings inclusive fitness benefits to a cheat but costs to a co-operator when cooperation is directed towards a cheat rather than an intended recipient (3). In a mixed population of cheats and non-cheats, cheats should fare better, and non-cheats should fare worse, than each would do in separate, homogeneous populations where the cheats have no one to cheat, and co-operators are not cheated upon.

Cooperation: a behaviour that benefits another individual that has been favoured by selection as a result of that beneficial effect (2).

Germline: the cells that connect generations of organisms through sexual reproduction (4). This refers to the gametes themselves, and to the progenitor germline stem cells that produce the gametes.

Individual: An entity that has undergone a major evolutionary transition: any constituent parts cannot reproduce outside of this entity, and there is low internal conflict compared to among individual conflict (5).

Major evolutionary transition: A major evolutionary transition in individuality is defined by two conditions (5). First, entities that were capable of independent replication before the transition can replicate only as part of a larger unit after it. Second, there is a relative lack of within-group conflict so that the larger unit can be thought of as a fitness-maximizing individual in its own right (6). Maynard-Smith and Szathmary identified stepwise increases in complexity that are referred to as major evolutionary transitions: the formation of genomes from independent replicators; the evolution of a DNA-RNA-protein replication system rather than just RNA; the formation of eukaryotes from proto-eukaryote engulfing proto-mitochondria; single cells joining to form multicellular organisms; and, in some insects, the formation of colonies from individual multicellular organisms.

Modularity: A modular organism is an organism built from repeating parts that are at least semi-autonomous, and can collect or produce their own sustenance and reproduce independently of other modules, although they may still rely on shared structures or resources. The branches of a tree, for example, are supported by the same trunk and root system, but each branch has leaves that collect energy and can produce reproductive flowers.

Pluripotent (plenipotent) cells: cells that are able to produce progeny that contribute to both germline and somatic cell (7). Pluripotent cells differ from totipotent cells, as they may not be able to produce all cell types, such as the interstitial cells of *Hydra* that produce both somatic cells and germline cells, but cannot produce all somatic cell types (8).

Relatedness: the probability that individuals share a gene that is identical by descent (9, 10). Relatedness should be considered at the locus for the social trait, rather than across a whole

genome, although relatedness is often the same across a genome. See box 2 of the main text for a more thorough discussion.

Selfish: an individual or a trait that benefits the individual expressing it, but comes at a cost to other individuals or the group (2).

Soma: the cells that are not the germline, they specialise on non-reproductive functions such as motility, resource capture, etc. In ‘typical’ animals, these cells die between generations and pass on no genes (4).

Unitary: In contrast to modular organisms, the parts of a unitary organisms are completely interdependent, we have some organs to collect food, and others that are exclusively reproductive. No part can survive or reproduce without the others: your arm relies on your gut to collect energy from food, and your reproductive organs for reproduction.

Unanswered questions in the evolution of the Metazoa

- How strong does within-organism selection need to be to overwhelm between-organism selection?

- Is there within-organism selection, and what effect does this have on the organism?

- Does within-organism variation persist through multiple generations?

- How frequent must a zygotic bottleneck be to maintain sufficiently high relatedness?

- What was the first Metazoan like? Are asexually reproducing animals with no obvious germline segregation derived?

- Does a strict germline facilitate increased complexity?

- Do germlines derived from pluripotent progenitors still behave as germlines (ie. lower mutation rates)?

- How much diversity is present within an individual? How does this vary with mode of reproduction?

- Is within-individual diversity detrimental to an obligately multicellular organism?

REFERENCES

1. A. Grafen, Formal Darwinism, the individual-as-maximizing-agent analogy and bet-hedging. *Proceedings of the Royal Society of London. Series B: Biological Sciences* **266**, 799-803 (1999).
2. S. A. West, A. S. Griffin, A. Gardner, Social semantics: altruism, cooperation, mutualism, strong reciprocity and group selection. *J Evol Biol* **20**, 415-432 (2007).
3. M. Ghoul, A. S. Griffin, S. A. West, TOWARD AN EVOLUTIONARY DEFINITION OF CHEATING. *Evolution* **68**, 318-331 (2014).
4. A. Weismann, *The Germ-plasm: a theory of heredity. Translated by W. Newton Parker and Harriet Rönnfeldt* (Scribner, New York, 1893).
5. S. A. West, R. M. Fisher, A. Gardner, E. T. Kiers, Major evolutionary transitions in individuality. *Proceedings of the National Academy of Sciences* **112**, 10112 (2015).
6. A. F. Bourke, *Principles of social evolution* (Oxford University Press, 2011).
7. M. L. Condic, Totipotency: What It Is and What It Is Not. *Stem Cells and Development* **23**, 796-812 (2013).
8. T. C. G. Bosch, C. N. David, Stem cells of Hydra magnipapillata can differentiate into somatic cells and germ line cells. *Developmental Biology* **121**, 182-191 (1987).
9. W. D. Hamilton, The genetical evolution of social behaviour. I. *J Theor Biol* **7**, 1-16 (1964).
10. W. D. Hamilton, The genetical evolution of social behaviour. II. *J Theor Biol* **7**, 17-52 (1964).
